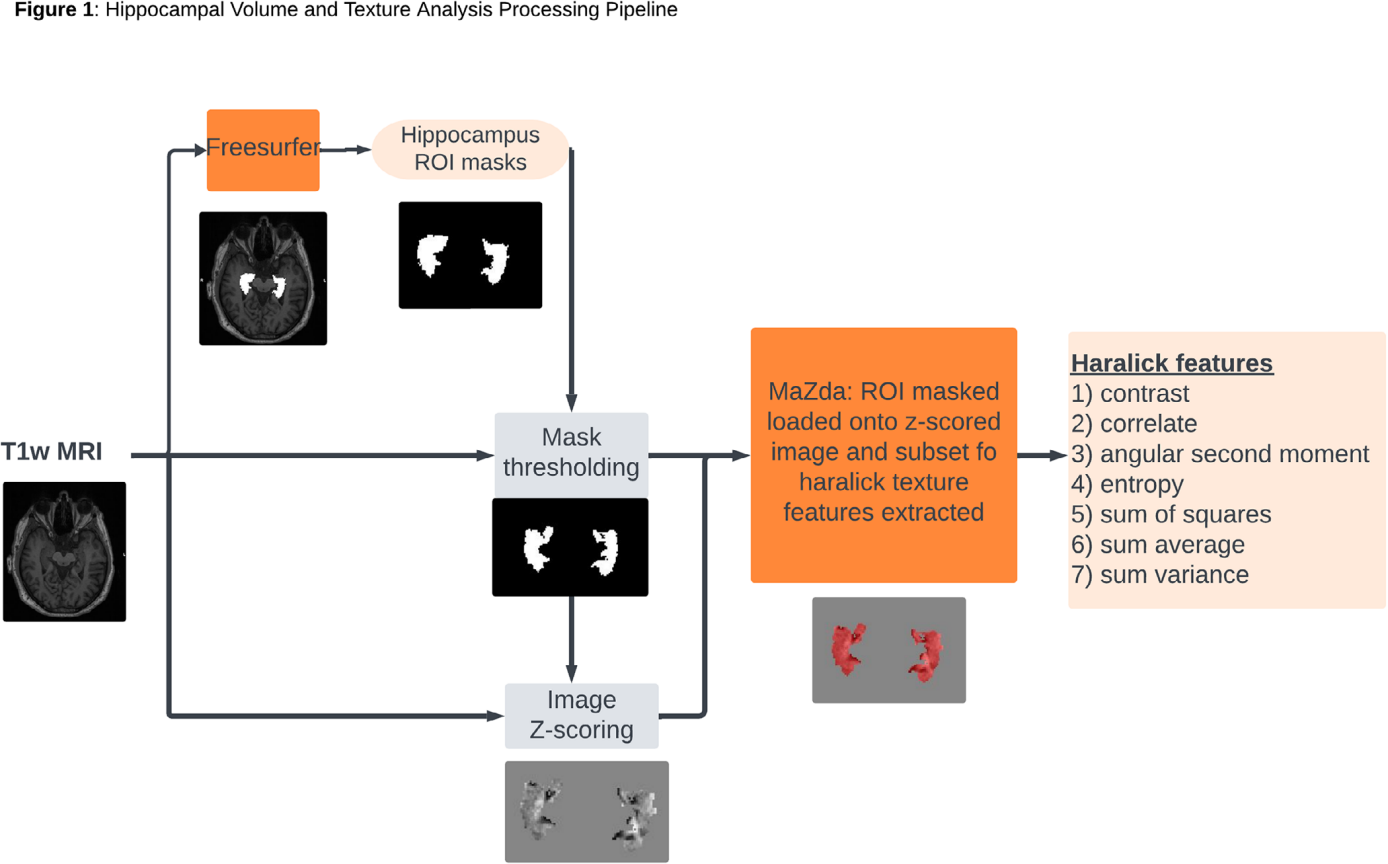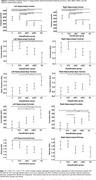# Texture analysis as a biomarker for Alzheimer's disease

**DOI:** 10.1002/alz70856_107254

**Published:** 2026-01-08

**Authors:** Y Mukish M Yelanchezian, Catherine A Morgan, Reece P Roberts, Tracy R Melzer, Ian J Kirk, Kiri L Brickell, Nicholas J Cutfield, Campbell J Le Heron, John C Dalrymple‐Alford, Tim J Anderson, Lynette J Tippett, New Zealand‐Dementia prevention clinic

**Affiliations:** ^1^ School of Medicine, University of Auckland, Auckland, New Zealand; ^2^ School of Psychology, The University of Auckland, Auckland, New Zealand; ^3^ Centre for Brain Research, The University of Auckland, Auckland, New Zealand; ^4^ NZ Brain Research Institute, Christchurch, New Zealand; ^5^ Department of Medicine, University of Otago, Christchurch, New Zealand; ^6^ Brain Health Research Centre, University of Otago, Dunedin, New Zealand; ^7^ Department of Neurology, Southern District Health Board, Dunedin, New Zealand; ^8^ Department of Medicine, University of Otago, Dunedin, New Zealand; ^9^ New Zealand Brain Research Institute, Christchurch, New Zealand; ^10^ University of Otago, Christchurch, New Zealand; ^11^ Te Kura Mahi ā‐Hirikapo | School of Psychology, Speech and Hearing, University of Canterbury, Christchurch, New Zealand; ^12^ The University of Auckland, Auckland, Auckland, New Zealand; ^13^ Centre for Brain Research, University of Auckland, Auckland, New Zealand

## Abstract

**Background:**

In Alzheimer's disease (AD), conventional magnetic resonance imaging (MRI) biomarkers have focused on macroscopic neurodegeneration via atrophy assessment, but measurable volume changes tend to occur later in the disease process. Recent studies suggest that texture analysis (TA) may capture microstructural changes associated with earlier AD pathology, potentially serving as a more sensitive biomarker for early identification of individuals at higher risk of progressing to dementia. TA is a mathematical method that quantifies spatial variation in grayscale values with rougher tissue texture represented by greater grayscale variation. Haralick features are a commonly used set of TA features that measure image heterogeneity, randomness, and smoothness.

**Aim:**

This study investigated whether Texture Analysis could distinguish between groups at‐risk of AD and classify convertors to dementia from non‐convertors.

**Method:**

231 individuals from the New Zealand‐Dementia Prevention Research Clinics participated (36 healthy controls, 61 with subjective cognitive decline, 46 with amnestic mild cognitive impairment (MCI), 63 with multidomain MCI and 25 with AD dementia). Using 3T MRI T1‐weighted images volume and a subset of 7 Haralick texture features was extracted from hippocampus for each participant (Figure 1). Groupwise differences in texture features within left and right hippocampal regions were analysed using MANCOVA and contrasted with groupwise volume differences analysed with ANCOVA. Machine‐learning was employed to test the performance of texture and volume features in distinguishing MCI converters to dementia from MCI non‐converters.

**Results:**

Volume, and five texture features (correlate, sum variance, sum average, contrast, and entropy texture), were significantly different between groups in both hippocampi, with a sixth feature, angular second moment, different across groups in the left hippocampus only (see Figure 2). Contrary to previous studies, classifying dementia conversion based on hippocampal volume outperformed classifiers based on texture (Accuracy=60.1%, Sensitivity=61.8%, Specificity=59.3% and Accuracy=56.1%, Sensitivity=57.9%, Specificity=54.6% respectively), though both classifiers only performed marginally better than chance.

**Conclusion:**

Our findings suggest that the utility of texture as an early biomarker of neural changes related to AD risk is not yet established. This highlights the necessity for additional research using different datasets to clarify the relationship between texture features, clinical impairment and progression to dementia.